# Induction of systemic anti-melanoma immunity through intratumoral TLR-7/8 activation

**DOI:** 10.1186/2051-1426-2-S3-P176

**Published:** 2014-11-06

**Authors:** Manisha Singh, Hiep Khong, Zhimin Dai, John P Vasilakos, Patrick Hwu, Willem W Overwijk

**Affiliations:** 1 Department of Melanoma Medical Oncology, The University of Texas MD Anderson Cancer Center, Houston, TX, USA; 2 3M Drug Delivery Systems Division, 3M Company, St. Paul, MN, USA

## Purpose

Intratumoral immune activation can induce systemic immunity and anti-tumor activity. Imiquimod is a cream-formulated, TLR-7 agonist that is FDA-approved for the treatment of non-melanoma skin cancers, but has limited activity against melanoma. In the current study, we studied the anti-tumor activity and mechanism of action of a novel injectable TLR 7/8 dual agonist, 3M-052, which remains at the site of injection to avoid systemic distribution.

## Experimental design

Mice bearing established B16 melanomas were treated intratumorally with 3M-052 or vehicle. The mechanistic contribution of individual cell types and molecules to the anti-tumor effect was determined using genetically engineered mice and antibody blockades. Immune cell infiltrates were analyzed by flow cytometry.

## Results

Intratumoral administration of 3M-052 generated systemic anti-tumor immunity and suppressed both injected and distant uninjected wild-type B16.F10 melanomas. Treated tumors showed increased level of CCL2 chemokines and CCL2 dependent infiltration of M1 phenotype-shifted macrophages which could kill tumor cells directly through production of nitric oxide. CD8^+ ^T cells, B cells, Type I IFN, IFN-g, and pDCs contributed to efficient tumor suppression whereas perforin, NK cells and CD4 T cells were not required.

## Conclusion

Induction of effective innate and tumor specific adaptive immunity by intratumoral treatment of TLR7/8 agonist, 3M-052 is a promising approach for the treatment of metastatic cancer.

